# Risk factors for new vertebral fractures after percutaneous vertebroplasty or percutaneous kyphoplasty in the treatment of osteoporotic vertebral compression fractures

**DOI:** 10.3389/fmed.2025.1514894

**Published:** 2025-01-22

**Authors:** Wencheng Yang, Kaiwei Zou, Xuping Lin, Yanfang Yang, Tianpei Chen, Xiuming Wu, Xiaomeng Wang, Qingjun Liu, Chunhui Huang, Wanhan Su

**Affiliations:** ^1^Department of Spine Surgery, Longyan First Affiliated Hospital of Fujian Medical University, Longyan, China; ^2^Department of Cardiology, Shengli Clinical Medical College of Fujian Medical University, Fuzhou, Fujian, China; ^3^Department of Orthopaedic, Affiliated Dongnan Hospital of Xiamen University, Zhangzhou, China

**Keywords:** osteoporotic vertebral compression fractures, senior citizen, new vertebral fractures, percutaneous vertebroplasty, percutaneous kyphoplasty, risk factors

## Abstract

**Object:**

This study aims to conduct a prospective analysis of patients with osteoporotic vertebral compression fractures (OVCF) who underwent percutaneous vertebroplasty (PVP) or percutaneous kyphoplasty (PKP), and further analyze the risk factors for new vertebral fracture following treatment.

**Methods:**

A prospective study was conducted from November 2020 to March 2022 at the First Hospital of Longyan City to select patients with OVCF who underwent treatment in the Department of Spinal Surgery. Data collection during the follow-up period focused on various factors that could potentially be associated with new vertebral fractures after PVP/PKP procedures. Patients were divided into two groups based on whether they experienced new vertebral fractures within two years after discharge: the new fracture group (*n* = 186) and the non-fracture group (*n* = 64), and statistical analysis was conducted accordingly.

**Results:**

All cases were followed up for 12 to 24 months, with an average of 14.7 months. Differential analysis revealed that age, diabetes, hemoglobin (HB), total protein (TP), serum albumin (ALB), b-C-terminal telopeptide of type I collage (β-CTX), 25-hydroxyvitamin D (25-OH-D3), number of fractured vertebrae, bone mineral density (BMD), regular exercise after discharge, anti-osteoporosis treatment after discharge, cross-sectional area (CSA), and fatty degeneration ratio (FDR) were associated with new vertebral fractures (all *P* < 0.05). Multivariate analysis showed that age (OR = 1.519, *P* = 0.032), diabetes (OR = 3.273, *P* = 0.048), and FDR (OR = 1.571, *P* = 0.027) were positively associated with the occurrence of new vertebral fractures, while bone mineral density (OR = 0.108, *P* = 0.044), 25-OH-D3 (OR = 0.871, *P* = 0.032), CSA (OR = 0.564, *P* = 0.009), regular postoperative exercise (OR = 0.259, *P* = 0.025), and osteoporosis treatment (OR = 0.291, *P* = 0.045) were negatively associated with the occurrence of new vertebral fractures.

**Conclusion:**

Patients with osteoporosis fractures who are older, have poor glycemic control, lower bone mineral density, lower levels of 25-OH-D3, weaker paraspinal muscles, and higher fat infiltration are at increased risk of new vertebral fractures after undergoing PKP/PVP. On the other hand, maintaining regular physical activity and adhering to osteoporosis treatment can help prevent new vertebral fractures.

## Introduction

Osteoporosis is a metabolic disorder distinguished by diminished bone strength and alterations in bone microstructure, resulting in heightened bone fragility (1). The condition is prevalent among the elderly. According to a Canadian multicentre osteoporosis study, 21.5% of men and 23.5% of women aged over 50 years exhibit at least one vertebral compression deformity (2). Osteoporotic vertebral compression fractures (OVCF) are fractures of the vertebral bodies caused by external forces acting on an osteoporotic bone structure. In elderly patients, the decline of various physiological functions often necessitates bed rest following a fracture, resulting in reduced mobility and further bone loss. Moreover, the fracture and the associated treatment can exacerbate bone loss, thereby increasing the risk of subsequent fractures (3, 4). Symptomatic OVCF can induce considerable pain and diminish a patient’s mobility, significantly affecting their quality of life (5).

Over the past few decades, percutaneous vertebroplasty (PVP) and percutaneous kyphoplasty (PKP) have been widely used to treat OVCF due to their rapid pain relief and functional improvement (6). However, new vertebral fractures after PVP/PKP remain a common issue. Previous studies have reported varying frequencies and timing of new vertebral fracture after treatment. Kim et al. (7) found that refracture occurred, on average, 3.4 months after PKP, with an incidence rate of 12.5%. Similarly, Chen et al. (8) conducted a retrospective analysis, reporting that 9.7% of 134 OVCF patients treated with PKP experienced re-collapse of the cemented vertebrae, resulting in severe back pain and dysfunction.

A study encompassing 178 patients with OVCF demonstrated that 68 patients (38.2%) encountered new vertebral fractures (9). The risk of experiencing a subsequent fracture within one year for osteoporotic vertebral fracture patients is approximately 2.7 times higher than for non-fracture patients (10). Previous studies have confirmed that patient characteristics such as gender, age, body mass index (BMI), and chronic diseases, as well as factors related to cement injection, may influence the occurrence of new vertebral fractures after PVP/PKP (11). Since osteoporosis is closely associated with lifestyle factors such as smoking, alcohol consumption, and physical activity, it is believed that these same factors may affect the incidence of postoperative new vertebral fractures.

Bone turnover markers are parameters that reflect the dynamic state of osteoporosis. By measuring serum bone metabolism markers, the bone turnover status in OVCF patients can be indirectly assessed. Several studies have explored the relationship between bone turnover markers and osteoporosis, including parathyroid hormone (PTH), C-terminal telopeptide of type I collagen (β-CTX), N-terminal propeptide of type I procollagen (PINP), osteocalcin (OC), serum 25-hydroxyvitamin D3 (25-OH-D3), and alkaline phosphatase (ALP) (12). In addition, some researchers have suggested that paraspinal muscle and fat infiltration are associated with OVCF (13, 14). However, no studies have comprehensively analyzed all of these potential risk factors. Therefore, the risk factors for new vertebral fractures after PVP/PKP in OVCF patients remain inadequately defined. Based on a large dataset from our hospital, this study aims to provide insights into the risk factors for new vertebral fractures after PVP/PKP in OVCF patients through differential analysis and multivariate regression analysis.

## Materials and methods

### Data sources

This study utilized a prospective design and selected 250 patients with OVCF who received surgical treatment in the Spinal Surgery Department of Longyan First Hospital from November 2020 to March 2022 as the research subjects. The patients were divided into a new fracture group and a non-fracture group based on whether they experienced another fracture within two years after discharge. There were 111 male and 139 female patients, with an age range of 60 to 80 years and an average age of 72.89 ± 7.93 years. Inclusion criteria were as follows: (1) 80 ≥ age ≥ 60 years; (2) diagnosed with OVCF based on diagnostic criteria and confirmed through X-ray/CT scan/MRI imaging (15); (3) initial admission for PKP or PVP treatment; (4) consent obtained from patients and their families for participation in the study; (5) availability of complete medical records, the willingness of patients to answer questions during follow-up, and provision of the necessary information for the study. The exclusion criteria were as follows: (1) neoplasms of the vertebral column; (2) history of vertebral fracture, spinal surgery, and low back soft tissue injury or surgery; (3) any other comorbidity or chronic diseases that could significantly affect bone or soft tissue metabolism (for example liver and kidney disease, chondromalacia, thyroid disorders, ankylosing spondylitis, diffuse idiopathic skeletal hyperostosis, or connective tissue disease); (4) history of certain drug use (hormonal drugs, anti-osteoporosis drugs, or diet pills); (5) severe cardiopulmonary diseases or coagulation dysfunction; and (6) incomplete clinical data. This study has been approved by the Medical Ethics Committee of Longyan First Hospital (L2022005). All participants in the study received written and oral information prior to giving written consent, and the study was performed following the Declaration of HELSINKI.

### Surgical procedure

Surgical procedure PVP/PKP was performed within three days of hospitalization for all patients. Every operation was conducted by the specified spinal surgeons with sufficient clinical experience, and the skills designed by Garfin et al. (16). The patients first placed in a prone position on the operating table were administered under local anesthesia (2% lidocaine). With the guidance of two single-plane mobile C-arms, the anterior–posterior and lateral views of the fractured vertebra were confirmed. After incision of the skin, two 11-gauge needles were placed parallel to the superior and inferior edges of the pedicle, percutaneously into the anterior part of the vertebral body with a transpedicular or perpendicular approach. For vertebrae with excessive anterior compression or significant kyphotic deformity, PKP surgery was performed. Under the guidance of a C-arm X-ray machine, a balloon was inserted, and the pressure was gradually increased to 200 PSI. Once the balloon was fully expanded and satisfactory vertebral height restoration was achieved, the balloon was removed. The injection of polymethylmethacrylate (PMMA) cement (Stryker, Kalamazoo, USA) into the fractured vertebral body was ceased until the cement reached the posterior one-fourth of the body or if PMMA extravasated outside the bone. The volume of bone cement inserted during the operation for each vertebra was recorded. Thoracic-lumbosacral orthosis was supplied to all patients for one month, and osteoporotic medications were used postoperatively.

### Study methods

Based on the electronic medical records system of elderly patients with OVCF admitted to the hospital, relevant patient information was obtained. We employed differential analysis and logistic regression analysis to identify the influencing factors for new vertebral fractures after discharge.

The following information was recorded for each patient: basic demographic data (gender, age, height, and weight), adverse lifestyle habits (smoking, alcohol consumption), presence of chronic diseases (hypertension, diabetes, coronary atherosclerosis), preoperative bone mineral density (BMD), bone metabolism-related markers, blood biochemical indicators, number of vertebral fractures, amount of bone cement injected per vertebra, the condition of cement leakage, postoperative exercise, and adherence to anti-osteoporosis treatment. During each postoperative follow-up, patients were asked whether they followed the doctor’s advice for exercise and whether they received osteoporosis treatment. Exercise was defined as aerobic activities, including brisk walking, jogging, dancing, swimming, and hiking, aimed at increasing heart rate for at least 30 min, at least four times per week. Osteoporosis treatment medications included calcitonin and bisphosphonates. According to the treatment plan prescribed at discharge, patients adhered to the medication regimen (including calcitonin and bisphosphonate used in our hospital) for at least 12 months during the follow-up period.

### Image analyses

**FIGURE 1:**
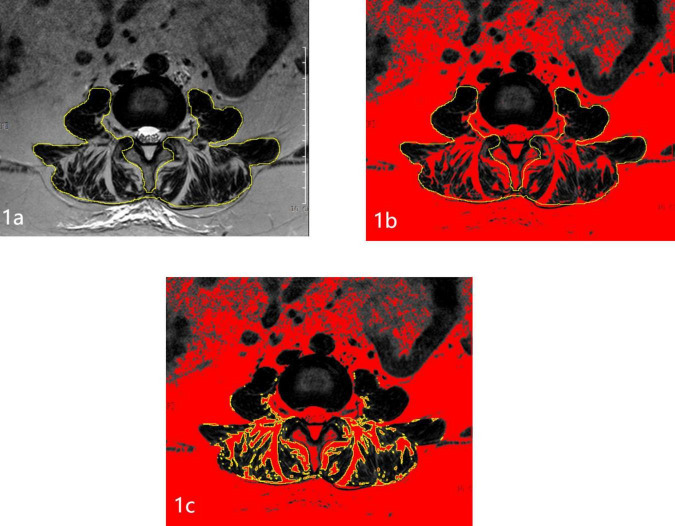
Schematic diagram of measuring paraspinal muscles and fat on MRI. **(A)** Measurement of the paraspinal muscle area. **(B)** Red represents all adipose tissue. **(C)** The intersection of the CSA and adipose tissue indicates the paraspinal FA.

Magnetic resonance imaging (MRI) examinations were completed using the 3.0T (Siemens Healthineers, Erlangen, Germany) scanner. T2-weighted images, parallel to the inferior endplate of the L3 vertebral body, were selected for analysis. The cross-sectional area (CSA) of the bilateral multifidus and erector spinae, and vertebral body size (VB) were separately outlined with the graphic cursor and measured on images using the hospital PACS digital imaging system (Figure 1). The fatty degeneration ratio (FDR) of the paraspinal muscle was analyzed and calculated using the ImageJ software for Windows (ImageJ version 1.53k, National Institutes of Health, Bethesda, MD, USA). Using yellow lines in ImageJ, the CSA was outlined and labeled. The pseudocolor tool in ImageJ was employed to identify adipose tissue regions, which were highlighted in red. The intersection between the CSA and the red adipose regions represented the paraspinal fatty area (FA). The FA obtained was then divided by the previously measured CSA to calculate the FDR.

### Statistical analysis

Statistical Methods Continuous data are expressed as means ± standard deviations, and the independent samples *t*-test was used for comparison between groups. Categorical data were analyzed by a chi-square test. A univariate analysis was used to identify potential influencing factors for new vertebral fracture after PVP/PKP. Multivariable logistic regression analysis was performed using the variables that showed statistical significance in the differential analysis. All statistical analyses were performed using SPSS Statistics for Windows, version 23.0 (IBM Corp, Armonk, NY, USA). A *P*-value of < 0.05 was considered statistically significant.

## Results

**FIGURE 2:**
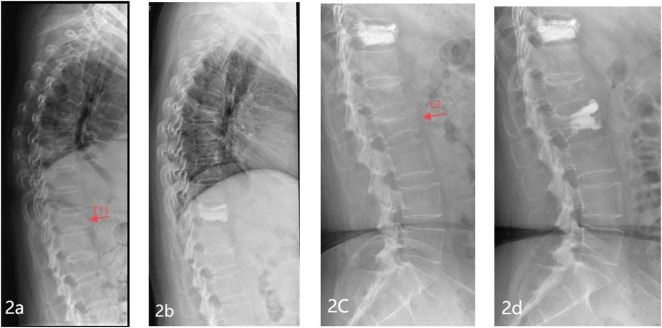
Representative images of a typical case of new vertebral fracture in a patient with OVCF after PVP/PKP. **(A)** Preoperative lateral X-ray of the thoracic spine showing a T11 vertebral compression fracture; **(B)** lateral X-ray of the thoracic spine 1 day post-operation showing T11 filled with bone cement; **(C)** lateral X-ray of the lumbar spine 1 year post-operation showing a new vertebral compression fracture at L2; **(D)** Lateral X-ray of the lumbar spine 1 day post-operation showing L2 filled with bone cement.

All cases were followed up for 12 to 24 months, with an average of 14.7 months. The study comprised 250 patients, among whom 64 experienced new vertebral fractures two years after follow-up, while 186 remained fracture-free (25.60%, 64/250) (Representative case shown in Figure 2). There were no statistically significant differences observed between the new fracture group and the non-fracture group concerning gender, BMI, habit (smoking/alcohol consumption), hypertension, coronary heart disease, red blood cell (RBC), white blood cell (WBC), platelet, uric acid (UA), blood glucose (Glu), triglyceride (TG), low-density lipoprotein cholesterol (LDL-C), serum calcium, serum phosphorus, ALP, OC, PINP, fracture site, PKP, the average volume of bone cement, cement leakage (*P* > 0.05). However, statistically significant variances (*P* < 0.05) were noted between the new fracture group and the non-fracture group in terms of age, diabetes, hemoglobin (HB), total protein (TP), serum albumin (ALB), total cholesterol (TC), high-density lipoprotein cholesterol (HDL-C), β-CTX, PTH, 25-OH-D3, number of fractured vertebrae, BMD, exercise regularly after discharge, anti-osteoporosis after discharge. Please refer to Table 1 for detailed information.

**TABLE 1:** Baseline characteristics of new fracture group and non-fracture group.

Variables	New fracture group (*n* = 64)	Non-fracture group (*n* = 186)	*P*-value
Gender (male/female)	24/40	87/99	0.198
Age (year)	75.64 ± 8.01	70.28 ± 7.94	0.021*
BMI (kg/m^2^)	22.04 ± 2.15	21.88 ± 2.86	0.824
Smoking (Yes/No)	22/42	42/142	0.069
Alcohol consumption (Yes/No)	16/48	36/150	0.337
Hypertension (Yes/No)	23/41	56/130	0.387
Diabetes (Yes/No)	20/44	44/142	0.032*
Coronary heart disease (Yes/No)	18/46	38/148	0.203
RBC (×10^12^/L)	4.34 ± 0.38	4.51 ± 0.43	0.151
HB (g/L)	114.32 ± 11.36	122.23 ± 13.96	0.031*
WBC (×10^9^/L)	7.75 ± 1.48	7.25 ± 1.26	0.207
Platelet (×10^9^/L)	237.24 ± 51.94	223.89 ± 53.51	0.577
TP (g/L)	61.69 ± 4.31	65.64 ± 4.43	0.002*
ALB (g/L)	37.36 ± 3.66	40.88 ± 4.69	0.005*
UA (umol/L)	334.40 ± 31.72	345.17 ± 41.33	0.291
Glu (mmol/L)	5.65 ± 0.73	5.85 ± 0.84	0.398
TC (mmol/L)	5.34 ± 0.33	5.22 ± 0.38	0.229
TG (mmol/L)	1.47 ± 0.28	1.57 ± 0.26	0.163
HDL-C (mmol/L)	1.31 ± 0.25	1.19 ± 0.24	0.107
LDL-C (mmol/L)	3.32 ± 0.47	3.41 ± 0.44	0.506
Serum calcium (mmol/L)	2.27 ± 0.19	2.33 ± 0.17	0.248
Serum phosphorus (mmol/L)	1.14 ± 0.19	1.11 ± 0.20	0.582
ALP (U/L)	80.64 ± 10.27	82.21 ± 10.82	0.604
β-CTX (ng/ml)	0.80 ± 0.15	0.67 ± 0.14	0.003*
OC (ng/ml)	23.38 ± 8.41	21.06 ± 7.73	0.315
PINP (ng/ml)	58.52 ± 9.51	56.88 ± 14.17	0.633
PTH (ng/ml)	50.36 ± 9.41	56.72 ± 10.48	0.029*
25-hydroxyvitamin D (nmol/L)	33.16 ± 10.01	41.81 ± 12.13	0.008*
Site (Thoracic /Lumba)	24/40	64/122	0.655
Number of fractured vertebrae			0.039*
1	38	136	
> 1	26	50	
Percutaneous kyphoplasty			0.539
Yes	44	120	
No	20	66	
The average volume of bone cement	4.48 ± 0.92	4.62 ± 1.02	0.635
Cement leakage			0.771
Yes	10	32	
No	54	154	
BMD (T-score)	−3.74 ± 0.74	−3.26 ± 0.63	0.015*
Exercise regularly			0.002*
Yes	19	96	
No	45	90	
Anti-osteoporosis			0.001*
Yes	14	108	
No	50	78	

Asterisk symbol (*) indicates *p*-value < 0.05, meaning a statistically meaningful. BMI, body mass index; RBC, red blood cell; HB, hemoglobin; WBC, white blood cell; TP, total protein; ALB, serum albumin; UA, uric acid; Glu, blood glucose; TC, total cholesterol; TG, triglyceride; HDL-C, high-density lipoprotein cholesterol; LDL-C, low-density lipoprotein cholesterol; ALP, alkaline phosphatase; β-CTX, b-C-terminal telopeptide of type I collagen; OC, osteocalcin; PINP N-terminal propeptides of type I procollagen; PTH, parathyroid hormone, BMD, bone mineral density, PKP, percutaneous kyphoplasty.

### Magnetic resonance imaging measurements

The VB of the two groups were (15.40 ± 2.36, 16.16 ± 2.44) cm^2^, with no statistically significant difference. The CSA of the two groups were (30.36 ± 3.57, 33.84 ± 4.18) cm^2^, and the difference was statistically significant (*P* = 0.003). The FDR of the two groups were (32.32 ± 4.58, 29.48 ± 4.54)%, and the difference was statistically significant (*P* = 0.032). Please refer to Table 2 for detailed information.

**TABLE 2:** Comparison of MRI imaging measurements between the two groups.

Variables	New fracture group (*n* = 44)	Non-fracture group (*n* = 106)	*P*-value
CSA (cm^2^)	30.36 ± 3.57	33.84 ± 4.18	0.003*
VB (cm^2^)	15.40 ± 2.36	16.16 ± 2.44	0.269
FA (cm^2^)	10.66 ± 1.86	9.68 ± 2.13	0.089
FDR (%)	32.32 ± 4.58	29.48 ± 4.54	0.032*

CSA, cross-sectional area; VB, vertebral body size, FA, fatty area; FDR, fatty degeneration ratio.

*Indicates *p*-value < 0.05, meaning a statistically meaningful.

### Multivariate logistic regression analysis

Based on the results of the differential analysis, variables with a significant probability of *P* < 0.05 were included in the subsequent multivariable logistic regression analysis. Therefore, the sixteen variables, including Age, Diabetes, HB, TP, ALB, HDL-C, β-CTX, PTH, CSA, VB, FDR, 25-OH-D3, Number of fractured vertebrae, BMD, Exercise regularly after discharge, Anti-osteoporosis after discharge, were included in the multivariate Logistic regression model. Multivariate analysis showed that age (OR = 1.519, *P* = 0.032), diabetes (OR = 3.273, *P* = 0.048), and fat infiltration (OR = 1.571, *P* = 0.027) were positively associated with the occurrence of new vertebral fractures (B > 0), while bone mineral density (OR = 0.108, *P* = 0.044), 25-OH-D3 (OR = 0.871, *P* = 0.032), paraspinal muscle area (OR = 0.564, *P* = 0.009), regular postoperative exercise (OR = 0.259, *P* = 0.025), and osteoporosis treatment (OR = 0.291, *P* = 0.045) were negatively associated with the occurrence of new vertebral fractures (B < 0). The specific analysis results are shown in Table 3.

**TABLE 3:** Risk factors related to the new vertebral fracture after PVP/PKP in OVCF patients (Multivariate logistic regression analysis).

Clinical parameters	B value	SE value	Wald value	*p*-value	OR value	Odds ratio 95% CI
Age (year)	0.413	0.462	5.327	0.032*	1.519	1.046∼1.852
Diabetes	1.186	0.601	3.897	0.048*	3.273	1.008∼10.621
HB (g/L)	−0.028	0.035	0.629	0.428	0.973	0.909∼1.041
TP (g/L)	−0.152	0.119	1.635	0.201	0.859	0.680∼1.085
ALB (g/L)	−0.065	0.096	0.453	0.501	0.937	0.777∼1.131
HDL-C (mmol/L)	0.015	1.592	0.001	0.993	1.015	0.045∼23.009
β-CTX (ng/ml)	1.665	2.213	0.567	0.452	5.288	0.069∼404.335
PTH (ng/ml)	−0.077	0.046	2.810	0.094	0.926	0.846∼1.013
CSA (cm2)	−0.572	0.219	6.803	0.009*	0.564	0.367∼0.867
VB (cm^2^)	−0.309	0.186	2.779	0.096	0.734	0.501∼1.056
FDR (%)	0.451	0.205	4.871	0.027*	1.571	1.052∼2.345
25-OH-D3 (nmol/L)	−0.138	0.064	4.581	0.032*	0.871	0.768 0.988
Number of fractured vertebrae	0.495	0.578	0.735	0.391	1.641	0.529∼5.093
BMD (T-score)	−2.230	1.107	4.057	0.044*	0.108	0.012 0.942
Exercise regularly	−1.350	0.604	4.992	0.025*	0.259	0.079 0.847
Anti-osteoporosis	−1.233	0.616	4.004	0.045*	0.291	0.087 0.975

HB, hemoglobin; WBC, white blood cell; TP, total protein; ALB, serum albumin; UA, uric acid; Glu, blood glucose; TC, total cholesterol; TG, triglyceride; HDL-C, high-density lipoprotein cholesterol; LDL-C, low-density lipoprotein cholesterol; ALP, alkaline phosphatase; β-CTX, b-C-terminal telopeptide of type I collagen; OC, osteocalcin; P1NP, N-terminal propeptides of type I procollagen; PTH, parathyroid hormone, BMD, bone mineral density; CSA, cross-sectional area; FDR, fatty degeneration ratio; VB, vertebral body size.

*Indicates *p*-value < 0.05, meaning a statistically meaningful.

### ROC curve and results

**FIGURE 3:**
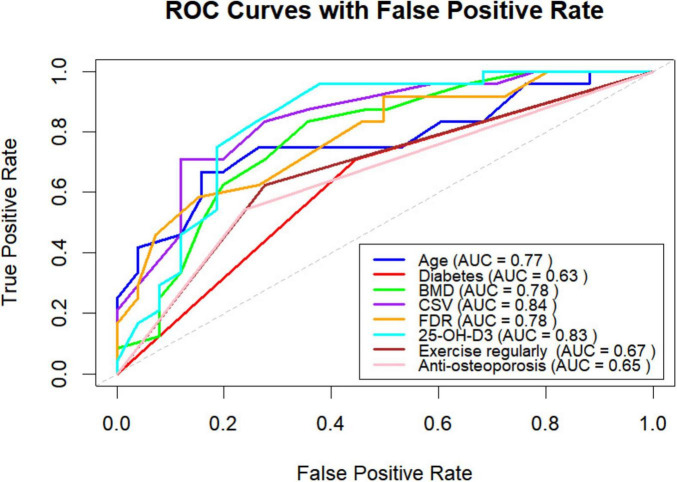
ROC curves of the selected variables after screening.

The receiver operating characteristic (ROC) curves for the variables of age, diabetes, CSA, FDR, 25-OH-D3, BMD, regular exercise after discharge, and anti-osteoporosis treatment after discharge are shown in Figure 3. The figure presents the cutoff values, specificity, sensitivity, positive and negative predictive values, and diagnostic efficiency for each factor. Among these variables, CSA demonstrated the highest AUC for predicting new vertebral fractures (0.84, 95% CI: 0.7902–0.8944). Both CSA and 25-OH-D3 had AUC values exceeding 0.8, while age, FDR, and BMD had AUC values greater than 0.7.

## Discussion

PVP and PKP are widely used for the treatment of osteoporotic thoracolumbar fractures due to their minimally invasive nature and rapid pain relief (17, 18). However, new vertebral fractures are a common long-term complication following these procedures (19, 20), and there is still considerable debate regarding the associated risk factors. Through differential analysis and multivariable logistic regression analysis, this study identified advanced age, diabetes, and fat infiltration as independent risk factors for new vertebral fractures after PVP/PKP in osteoporotic thoracolumbar fractures, while CSA, 25-OH-D3, regular exercise after discharge, and anti-osteoporosis treatment after discharge were found to be protective factors. In this study, the incidence of new vertebral fractures after PVP/PKP was 25%, slightly higher than in previous studies (21–23), which may be due to the older age of the study population and the longer follow-up period.

The incidence of subsequent fractures in elderly patients with OVCF post-discharge significantly exacerbates the decline in patients’ quality of life (24). Subsequent fractures not only subject patients to heightened levels of pain and inconvenience in daily life but may also exacerbate the initial spinal deformity, thereby amplifying the risk of paralysis and mortality (25). Hence, acquiring a comprehensive understanding of and effectively intervening in factors associated with subsequent fractures post-discharge is of paramount importance for enhancing patients’ quality of life and averting future fracture occurrences (26).

This study found that advanced age is an independent risk factor for new vertebral fractures after PVP/PKP in osteoporotic thoracolumbar fractures, consistent with the findings of Zhang et al. (23). The possible reasons are as follows: First, with aging, bone mass gradually decreases. Warden et al. (27) suggested that in older adults, reduced calcium intake and increased excretion lead to secondary hyperparathyroidism and a decrease in calcium ions within the bones. Additionally, Russell and Kahn (28) proposed that reduced sex hormone production in the elderly, along with increased oxidative stress, inhibits osteoblast activity, further exacerbating osteoporosis. Second, reduced physical activity in older adults further worsens osteoporosis. Finally, the elderly often have poorer self-care ability, making them more prone to falls and other accidents.

There is substantial and growing evidence that diabetes is associated with osteoporosis, despite bone mineral density usually remaining normal. This study found that diabetes is an independent risk factor for new vertebral fractures after PVP/PKP in osteoporotic thoracolumbar fractures. Given that diabetic patients tend to have worse fracture outcomes (including higher mortality rates), careful consideration should be given to fracture prevention management for this highly vulnerable group (29). Osteoporosis is emerging as a complication of diabetes. Compared to the general population, individuals with diabetes have a significantly higher risk of fractures. Osteoporosis in diabetes results from complex, poorly understood mechanisms at the cellular level, involving vascular, inflammatory, and mechanical disruptions (30). Both intrinsic bone factors, such as accumulation of advanced glycation end products, low bone turnover, and changes in bone microstructure, and extrinsic factors, such as hypoglycemia induced by treatment, diabetic peripheral neuropathy, muscle weakness, visual impairment, and certain glucose-lowering medications that affect bone metabolism, may contribute to impaired bone strength and an increased risk of fragility fractures (31). Bone mineral density serves as a crucial indicator of bone strength, intricately linked to the pace and quality of fracture healing (32). Low bone mineral density commonly signifies diminished internal bone tissue strength, compromised structural integrity, decreased load-bearing capacity, and stability, rendering bones more susceptible to injury or collapse even under minor external forces (33).

Previous research by Lee et al. (11) found that patients with lower BMD had a higher risk of new vertebral fractures. Similarly, Wang et al. (34) identified BMD as an independent risk factor for new vertebral fractures following PKP. Li et al. (35) demonstrated that low BMD increases the risk of new vertebral fractures after PVP or PKP. In a four-year follow-up study of patients with OVCF who underwent PVP or PKP, He et al. (36) found that BMD was the only risk factor for new vertebral fractures. Consistent with these previous studies, this study also confirms that BMD is an independent predictor of new vertebral fractures in OVCF patients post-surgery.

25-OH-D assumes a pivotal role in bone remodeling by fostering enhanced absorption of calcium and phosphorus in the intestines, augmenting bone metabolism, activating osteoblasts for new bone matrix formation, bolstering muscle contraction function, and expediting bone repair and healing processes (37). Postoperative patients with insufficient vitamin D3 levels may experience poor bone healing and further decline in bone mineral density, increasing the risk of new vertebral fractures. Vitamin D3 deficiency can lead to impaired bone mineralization, making bone tissue fragile. Several studies have shown that vitamin D3 supplementation significantly reduces the rate of new vertebral fractures in patients with osteoporosis (12). For instance, a randomized controlled trial found that patients who regularly supplemented with vitamin D3 had a significantly lower incidence of new vertebral fractures within one year post-surgery compared to those who did not supplement. As a crucial factor in maintaining bone health, vitamin D3 has a significant impact on reducing the risk of new vertebral fractures in postoperative osteoporosis patients (38). Future research could further explore its mechanisms of action and optimize supplementation strategies to improve clinical outcomes.

This study confirms that the decline in lumbar muscle mass and muscle fat degeneration are important risk and predictive factors for lumbar vertebral fractures. Previous studies have referred to this muscle condition as sarcopenia, characterized by the progressive loss of muscle mass and the associated reduction in strength and function with aging (39, 40). While the concept of sarcopenia is widely accepted, there is no consensus on how to measure and quantify it. To accurately analyze the qualitative and quantitative differences in lumbar muscle between groups, MRI imaging was used for measurement. Additionally, in this study, MRI-based measurements were identified as independent and sensitive predictors of secondary fracture risk. As shown in the study results, the fracture group exhibited significantly higher FDR and CSA. Similar findings were reported by Tokeshi et al. (14), who confirmed the phenomenon in their study, which included all OVCF participants regardless of injury mechanism. Jeon et al. (13) also reported that paraspinal muscle fat degeneration is a risk factor for progressive vertebral compression in OVCF patients. Wang et al. (34) further identified sarcopenia as an independent predictor of osteoporotic vertebral compression fractures. Even after vertebral augmentation procedures for OVCF, sarcopenic patients face a higher risk of postoperative mortality (41). The paraspinal muscles are crucial for maintaining normal spinal alignment, and the loss of muscle mass and increase in fat degeneration severely impact spinal balance and muscle strength, leading to frailty and an increased risk of fragility fractures. This study indicates that FDR and CSA can predict the risk of fragility fractures in elderly osteoporotic patients, with their predictive power being independent of, and even equal to or greater than, BMD (T-score).

Patients who fail to adhere to regular exercise post-discharge and neglect consistent intake of anti-osteoporosis medications are at an elevated risk of subsequent fractures. Inactivity can result in reduced bone mineral density and diminished muscle strength, exacerbating osteoporosis and the susceptibility to falls. Consistent physical activity not only aids in preserving or augmenting bone mineral density but also fortifies muscle strength, coordination, balance, and flexibility, thereby diminishing the risk of instability and falls (42). Moreover, regular exercise contributes to sustaining the range of motion of joints and muscles, thereby mitigating the risk of fractures stemming from limited mobility. The results of this study indicate that regular exercise is a protective factor against new vertebral fractures following PKP/PVP. Physical activity has been shown to improve BMD in elderly osteoporotic patients after PKP. With the increasing trend of an aging population in China, the number of elderly patients with osteoporosis is on the rise. If elderly patients with OVCF do not receive anti-osteoporosis medication post-surgery, their bone fragility may worsen. Early rehabilitation guidance after discharge can promote functional recovery. Appropriate exercise can improve BMD, reduce bone loss, and help prevent osteoporosis. The mechanism of treatment involves hormonal regulation; aerobic exercise can lower calcium levels in the blood, inhibit thyroid hormone secretion, suppress bone resorption, and promote bone synthesis, significantly increasing BMD. Exercise also enhances neuromuscular function, improving muscle strength and body mass, which helps prevent bone loss, increases BMD and bone strength, and ultimately improves bone and functional outcomes in elderly patients with osteoporosis (43, 44). Some studies have also shown that exercise regulates neuroendocrine function, promotes calcium absorption and utilization, and reduces calcium loss from bones, effectively preventing osteoporosis (45). However, long-term lack of weight-bearing and muscle activity can result in significant calcium loss from bones, exacerbating osteoporosis.

Likewise, irregular adherence to anti-osteoporosis medications disrupts their therapeutic efficacy, diminishes bone protection, weakens bone structure, and heightens the susceptibility to fractures (46). Anti-osteoporosis medications are designed to manage the advancement of osteoporosis and fortify bone integrity. Non-compliance with the medication regimen impedes this process, hastens osteoporosis progression, and complicates the assessment of treatment efficacy and subsequent adjustment of treatment protocols. Recent research has revealed that patients who inconsistently adhere to anti-osteoporosis treatment face a 3.40-fold higher risk of subsequent fractures compared to those who consistently follow the prescribed medication regimen (47). Therefore, to effectively mitigate the risk of subsequent fractures, individuals with osteoporosis should participate in suitable and consistent exercise regimens and adhere to their anti-osteoporosis medication schedules post-discharge. This proactive approach is vital for preserving the health of bones, muscles, and joints (48, 49).

Nevertheless, several limitations persist within this study. Primarily, the study’s sample size is modest, inevitably introducing biases. Another limitation of this study is the insufficient sample size, which prevented the integration of various predictive factors to develop a new clinical prediction model for vertebral refracture. Furthermore, the clinical data were solely collected from the orthopedic department of our hospital, thus lacking comparative analysis with other centers. Hence, further large-scale, multicenter prospective studies are imperative to corroborate these findings.

## Conclusion

In conclusion, our findings highlight several critical risk factors associated with subsequent fractures in elderly patients with OVCF, including advanced age, low bone mineral density, low 25-OH-D levels, irregular post-discharge exercise, and inconsistent use of anti-osteoporosis medications. Notably, we emphasize that regular physical activity and strict adherence to osteoporosis treatment significantly reduce the risk of new vertebral fractures. These results underscore the importance of personalized interventions targeting modifiable factors to improve long-term outcomes in this vulnerable population.

## Data Availability

The raw data supporting the conclusions of this article will be made available by the authors, without undue reservation.
